# Dynamics of emotional reactions among Russian students during the COVID-19 pandemic

**DOI:** 10.1192/j.eurpsy.2022.1243

**Published:** 2022-09-01

**Authors:** I. Gorkovaya, D. Ivanov, Y. Aleksandrovich, V. Titova, V. Rozhdestvenskiy

**Affiliations:** Saint Petersburg State Pediatric Medical University, Department Of Psychosomatics And Psychotherapy, Saint Petersburg, Russian Federation

## Abstract

**Introduction:**

The pandemic of a new coronavirus infection can be considered as a long-term traumatic event. It is known that chronic stress is characterized by dynamics of emotional state caused by processes of adaptation and maladaptation.

**Objectives:**

Our study aimed to investigate the dynamics of depression, anxiety, and stress levels among Russian students during the COVID-19 pandemic.

**Methods:**

Data were collected from May to July 2020 (the first pandemic wave) and from October 2020 to April 2021 (the second wave). A total of 170 non-medical university students participated in the study. We used the DASS-21 to determine levels of depression, anxiety, and stress.

**Results:**

We found that during the first wave of the pandemic, 57 % of students showed no symptoms of depression, 77 % had no symptoms of anxiety, and 76 % showed no signs of stress. In the second wave, 50 % of students showed no depression, 65 % no anxiety and 67 % no stress. Analysis of mean values showed that the second pandemic wave provoked higher levels of anxiety (M = 3.32±4.25 vs M = 4.71±4.71, p < 0.05) and stress (M = 6.50±4.50 vs M = 7.99±4.97, p < 0.05) .

**Conclusions:**

The second wave of the new coronavirus pandemic provoked more severe emotional reactions among Russian students than the first. By these results we suggest that the duration of the pandemic harms the emotional state of the general population. Therefore, it is essential to develop and implement psychotherapeutic programs to restore the mental health of Russian citizens.

**Disclosure:**

No significant relationships.

